# Proceedings of New York Homœopathic Union

**Published:** 1888-06

**Authors:** Clarence Willard Butler

**Affiliations:** Secretary


### THE PROCEEDINGS OF THE NEW YORK HOMŒOPATHIC UNION.

The first meeting of the New York Homœopathic Union was held at the office of Dr. Edward Bayard, No. 8 West Fortieth Street, New York city, April 19th, 1888. The following circular was addressed to many physicians residing in and about the cities of New York and Brooklyn by the gentlemen whose names are appended:

NEW YORK, April 14th, 1888.

DEAR DOCTOR:—After consultation among a number of homœopathic physicians, living in New York and vicinity, it is deemed best to form an association for purposes of mutual support in the practice of our art, and especially in keeping alive the great truths promulgated by Hahnemann. The necessity of some action is apparent and needs no argument. Let us know each other not through a glass darkly, but face to face. We propose to meet at the residence of Edward Bayard, M. D., No. 8 West Fortieth Street, New York, where you are cordially invited to come, Thursday, April 19th, 1888, at eight o'clock in the evening, organize, decide on times and places of meeting, and then *all go to work.* Will you join us?

EDWARD BAYARD,
P. P. WELLS,
CLARENCE WILLARD BUTLER,
E. CARLETON.

In response the following physicians were present: Drs. E. Carleton, J. W. Thomson, J. F. Miller, B. Fincke, J. Van Evera, C. Williams, Samuel Lewis Eaton, E. Bayard, C. C. Howard, J. Finch, H. Hitchcock, B. L. B. Baylies, E. Rushmore, and Clarence Willard Butler. Communications expressing sympathy with the objects of the Society and desire for active membership were received from the following, who were unavoidably absent: Drs. P. P. Wells, Alice B. Campbell, R. Heber Bedell, and Phebe D. Brown. Dr. Carleton was chosen temporary Chairman, Dr. Butler, Secretary. Dr. Carleton briefly stated the objects of the Society to be the study of Homœopathy both in respect of its philosophy as a science and its practice as an art. Dr. Bayard spoke of the difficulty of individualization by physicians in large practice, and of the dangers that such physicians would become generalizers. If this occurs, the desire for a generalized *Materia Medica* necessarily follows, since such physicians have no time for study. The jewel Truth was only brought to light by hard work, sacrifices, and suffering. Societies with such objects as those of this Society are an imperative necessity, that these dangerous tendencies may be combated and Homœopathy preserved in its purity. It should be our endeavor to hold aloft the great light which Hahnemann separated from the darkness, and which busy and careless men are trampling in the dust. He advocated the simplest form of organization, and suggested as a name, the New York Homœopathic Union. Dr. J. W. Thomson suggested the name The New York Hahnemannian Society. Dr. B. L. B. Baylies thought that stress should be laid upon the word "Homœopathic," which covers the principal fact.

Dr. Fincke liked the name Homœopathic Union; Homœopathic, he said, was enough. The union would indicate the union of the homœopathic remedy and the dose. It is not enough that a remedy simply covers the symptoms, but it must be homœopathic to the nature of the disease as well. He then read some carefully selected extracts from the *Organon* in proof of this position. The informal meetings of earlier days, he said, had been most valuable to him. The larger societies with their machinery had "machined" out nearly all of Homœopathy and of profitable study.

Dr. Miller said that it had been stated that the Hahnemannian homœopaths were to the great body of the homœopathic profession as one to one hundred. For this reason he favored a distinctive title for the Society.

Dr. Bayard thought that this Society had nothing to do with the practice or belief of the majority of the profession. It was organized that its members might bring to it for the mutual improvement all the good, all the truth, they could. It should fight no battles and enter into no controversies.

Dr. Howard said that only physicians who were trying to practice Homœopathy in its purity, as we are, had any right to the honorable title of homœopathists. There was no need for any other name.

Dr. Hitchcock desired to know if it was thought advisable to have a pledge and an elaborate organization.

Dr. Bayard said, "The more simple the organization the better. We want no pledge."

Dr. Fincke thought all should be welcome. If men came in who were not in sympathy with the objects of the Society, our study of the *Organon* would soon drive them away.

Dr. Thomson still thought that the title, Hahnemannian Union, as a tribute to Hahnemann, was the one which should be chosen.

Dr. Miller moved the appointment of a committee of three on permanent organization. Carried.

Drs. Miller, Rushmore, and Butler were appointed.

Dr. Miller declined to serve, and Dr. Finch was appointed in his stead. Dr. Hitchcock moved that we proceed to the study of the *Organon*. Dr. Fincke read Section 1.

Dr. Carleton asked, "Have we not a duty, as homœopathic physicians, in teaching those who say they will do anything to help their patients?"

Dr. Fincke referred to Sections 2 and 3, which he read.

The Committee on Organization then reported the Constitution and By-laws, which were accepted.

The following officers were next elected:

President, Edward Bayard, M. D.

Vice-President, B. Fincke, M. D.

Secretary and Treasurer, Clarence Willard Butler, M. D.

Dr. Finch suggested that some portion of the *Organon* be read and discussed at each meeting.

Dr. Hitchcock asked Dr. Fincke if he would not bring over his translation of the *Organon* for comparisons with those already in print. This Dr. Fincke kindly consented to do.

Section 6 of the *Organon* was then read.

Dr. Hitchcock spoke of the importance of the history to the treatment of a case. He had a case of polypus in the throat. There were no symptoms which he could elicit. The history of a patient showed a menstrual disturbance twelve years before, which should have received Sepia. He now gave Sepia, and a few doses cured the polypus within two years.

Dr. Butler thought that the pathological anatomy should be studied more carefully with reference to therapeutics. From the nature of the case few provings would ever be pushed far enough to produce tissue change; but clinical experience would add valuable symptoms to our *armamentum medicorum* in this field, as it had in the purely subjective. The totality of the symptoms must include these tissue changes, and the more we know of them as indicators for drug exhibition, the greater our resources.

Dr. Thomson did not think these would ever have other than diagnostic or prognostic value.

Dr. Butler—They will always be secondary to the subjective symptoms, because less peculiar and uncommon.

Dr. Van Evera said that we know that drugs can produce such symptoms, and they must certainly be within the range of curative action of such drugs when arising from other causes.

Dr. Carleton warned against mere symptom covering, without careful examination, especially for the mechanical causes of disease. When these exist their removal will prove the only proper treatment. He instanced cystitis, dependent upon the presence of calculi in the bladder, and the many uterine symptoms which were frequently, promptly, and radically removed by operating for the laceration of the cervix, which was indirectly their cause.

Dr. Fincke called attention to the favorable results in the after treatment of surgical cases from homœopathic medication.

Dr. Bayard spoke of the possibility of cure of diseased conditions in their earlier manifestations which otherwise would eventually demand surgical treatment.

Dr. Fincke mentioned the case of a man who was poisoned by sumach, and suffered for a long time with symptoms referred to the urinary apparatus. He eventually passed a large calculus.

Dr. Thomson said that disease often takes on that type which had been prominent in the patient's ancestors.

Dr. Bayard related the case of a man who, after exposure, had most violent earache. The pain was very acute, was in and behind the ear, running down behind the neck, with much throbbing. He confessed with such a severe case he hesitated to trust to the common dandelion, but did, and Taraxacum cured brilliantly. He took occasion now to say that he thought it an honor to be made President of the New York Homœopathic Union. He believed that the Society had profitable work to do, and would do it. He suggested that each member bring his copy of the *Organon* to the meetings.

The study of the *Organon* for the next meeting will be Sections 5, 6, and 7.

Dr. Baylies referred to the repetition of doses, and said that he did not consider repetition of doses without intermediate examination of the patient homœopathic.

Dr. Bayard thought that in acute cases, where the direct action of the morbific force was strong and rapid, repetition was necessary.

Dr. Thomson agreed, and instanced a case where the patient, an old lady, was very suddenly taken very severely ill, and where the indicated remedy relieved in frequently repeated doses. He stated that in such cases he *had* to repeat frequently.

Drs. Finch and Butler could not understand how he knew that he must repeat if he had not tried the single dose.

Dr. Baylies instanced cases where a single dose of a high potency was followed by sharp aggravation, later by speedy recovery.

Dr. Butler advocated trying the single dose first; then a repetition of doses later if examination showed the remedy was well chosen and satisfactory results had not followed the single dose.

Dr. Hitchcock reported the following case as illustrating the rapidity of action of the well chosen remedy: Severe neuralgia of head and eye, worse on the right side; shooting pains with relief from heat. Magn-phos.$^{cm}$, one dose, relieved entirely in five minutes.

Dr. Baylies stated as his conviction that the single dose was especially indicated in treating cases of chills and fever. He also told of a case of diphtheria which had been cured by a single dose of Lac-caninum.

Dr. Bayard stated that the late Dr. Reissig was the discoverer of the value of Lac-caninum. Also that Dr. Reissig had a theory that substances which were easily digested and nutritious in large quantities, were very powerful poisons when triturated or potentized, that is, very minutely divided by these means. He had used potentized beef in small-pox with wonderful results. Dr. Bayard once recommended its use to a medical man in Boston, who gave it in frequently repeated doses of the 30th with the most terrible aggravation. Dr. Reissig made provings of these substances and used them in the true homœopathic way. The oyster was another of his remedies, one which he found useful in scrofulous cases. The potentized oyster, Dr. Bayard said, was the vilest smelling stuff he had ever encountered. Dr. Bayard paid a high tribute to Dr. Reissig's attainments both as a scientist and as a gentleman. Dr. Reissig, he said, was not a secretive man, but was sensitive, and did not publish either his provings or his cures, because he thought the profession was not yet ready for them.

Adjourned at 10.40 P. M.

CLARENCE WILLARD BUTLER,
*Secretary.*

